# The Role of Organ Sparing Approaches After Total Neoadjuvant Treatment in Rectal Cancer

**DOI:** 10.3390/cancers18010055

**Published:** 2025-12-24

**Authors:** Gianluca Rizzo, Vincenzo Tondolo, Luca Emanuele Amodio, Federica Marzi, Camilla Marandola, Donato Paolo Pafundi, Giuseppe De Rito, Claudio Coco

**Affiliations:** 1 Digestive and Colo-Rectal Surgery Unit, Ospedale Isola Tiberina Gemelli Isola, 00186 Rome, Italy; vincenzo.tondolo@fbf-isola.it (V.T.); federica.marzi.fw@fbf-isola.it (F.M.); camilla.marandola01@icatt.it (C.M.); 2 General Surgery 2 Unit, Fondazione Policlinico Universitario Agostino Gemelli IRCCS, 00168 Rome, Italy; donatopaolo.pafundi@policlinicogemelli.it (D.P.P.); giuseppe.derito01@icatt.it (G.D.R.); claudio.coco@unicatt.it (C.C.)

**Keywords:** total neoadjuvant treatment, rectal cancer, watch & wait, local excision, organ-sparing approach

## Abstract

Organ-preserving strategies have gained increasing relevance in the management of rectal cancer, driven by the improved ability of neoadjuvant therapies to induce major and complete tumor regression. The introduction of Total Neoadjuvant Therapy (TNT), delivered through induction and/or consolidation chemotherapy combined with radiotherapy, has substantially increased both pathological and clinical complete response rates. This progress has renewed interest in non-operative management—namely Watch-and-Wait (W&W)—and in local excision (LE) as potential alternatives to total mesorectal excision (TME). However, the W&W strategy raises important oncologic concerns, including a non-negligible rate of local regrowth—consistently reported at approximately 20–30%—which is associated with inferior distant metastasis-free survival and overall survival. These limitations underscore the inherent uncertainty in reliably defining a true clinical complete response. Within this context, LE may serve as a valuable diagnostic and therapeutic modality by confirming the pathological response, improving local control through removal of residual resistant tumor clones, and enabling more accurate stratification of patients suitable for organ preservation versus those requiring completion TME. Overall, while TNT has expanded the therapeutic opportunities for rectal preservation, LE appears to play a critical role in reducing the discordance between clinical and pathological assessment, thereby offering a more oncologically secure pathway toward organ preservation. This narrative review discusses the current role, benefits, and limitations of organ-preserving approaches after TNT in both locally advanced and early rectal cancer.

## Simple Summary

Advances in Total Neoadjuvant Therapy (TNT) have increased complete responses in rectal cancer, raising interest in organ-preserving approaches. The Watch-and-Wait (W&W) strategy can avoid surgery but carries a relevant risk of local regrowth, which may worsen long-term outcomes. Local excision (LE) offers an alternative that allows for confirmation of true tumor disappearance and removal of any residual cancer cells, providing greater oncologic safety. This review summarizes current evidence on W&W and LE after TNT to help guide safe and tailored organ-preservation strategies.

## 1. Introduction

The management of extraperitoneal rectal cancer typically requires a multidisciplinary approach. In patients with locally advanced rectal cancer (LARC), the combination of Total Mesorectal Excision (TME) and preoperative radiotherapy (RT) with concomitant chemotherapy (CRT) remains the gold standard of care [1]. This multimodal strategy significantly reduces the rate of local recurrence (LR) after TME, increases the likelihood of sphincter-preserving surgery due to the downsizing and downstaging effects of CRT, and achieves a meaningful rate of pathological complete response (pCR), an important prognostic indicator of oncological outcomes [2,3].

Despite these benefits, neoadjuvant CRT does not appear to improve distant recurrence rates or overall survival following TME in LARC [4]. To overcome this limitation, Total Neoadjuvant Therapy (TNT)—which incorporates additional chemotherapy (CT) before surgery—has been proposed. Two TNT modalities have been described: induction TNT (iTNT), where CT precedes CRT, and consolidation TNT (cTNT), where CT is administered after CRT. Both regimens are followed by surgical resection [5]. Early results from clinical trials indicate that TNT achieves higher clinical complete response (cCR) and pCR rates compared with standard CRT. A cCR is defined as the absence of detectable residual tumor on endoscopy or imaging, while a pCR corresponds to the absence of viable tumor cells on histopathological examination.

The increased frequency of complete responses, together with the short-term morbidity and often suboptimal long-term functional results associated with TME, has fueled growing interest in organ-preserving strategies, including non-operative management (NOM) and local excision (LE) [6,7,8,9]. Encouraged by the favorable outcomes observed in LARC, combined chemoradiation approaches have also been extended to selected early-stage low–mid rectal cancers (T2–T3 N0) with the goal of expanding opportunities for rectal preservation [10].

The objective of this narrative review is to evaluate the role of organ-sparing strategies—NOM and LE—in the era of TNT for rectal cancer, highlighting their advantages, limitations, and current controversies.

## 2. Rationale and Oncologic Outcomes of TNT in LARC

TNT was developed to improve the high rate (approximately 30%) of distant failure observed after standard CRT regimens. This failure rate may be related to poor compliance with adjuvant chemotherapy, often due to postoperative morbidity, treatment delays, and increased frailty following surgery. TNT aims to deliver both systemic chemotherapy and radiation therapy in the preoperative setting.

Several studies have compared TNT with standard CRT. The randomized RAPIDO and PRODIGE-23 trials demonstrated significant benefits of TNT in terms of disease-free survival (DFS), distant metastasis rates, and pCR compared with standard CRT [11,12,13,14,15]. In the RAPIDO trial, the TNT group achieved a significantly lower 3-year distant metastasis rate (20% vs. 26.8%; HR 0.69 [95% CI 0.54–0.90]; *p* = 0.0048) without a corresponding improvement in overall survival (OS) [11,12]. Conversely, the PRODIGE-23 trial reported significant advantages for TNT in both 7-year DFS (67.6% [95% CI 60.7% to 73.9%] vs. 62.5% [95% CI 55.6% to 68.6%] *p* = 0.048) and 7-year OS (81.9% [95% CI 75.8% to 86.6%] vs. 76.1% [95% CI 69.7% to 81.2%]; *p* = 0.033). However, the interpretation of these results is limited by the study design: neoadjuvant CT in the TNT arm replaced adjuvant chemotherapy in the control arm, and irinotecan was added only in the TNT arm. Thus, it remains unclear whether the OS benefit was attributable to treatment sequencing, irinotecan use, or both. Additionally, the FOLFIRINOX regimen used in PRODIGE-23 is rarely adopted in clinical practice due to its significant toxicity [13,14].

Long-term oncologic outcomes after TNT have also been evaluated in systematic reviews and meta-analyses. In the meta-analysis by Petrelli et al. [8], which included 28 studies and 3579 patients, outcomes of 2688 patients treated with TNT were compared with 891 receiving standard CRT. TNT showed improved—but not statistically significant—DFS and a significantly better OS. The pooled 1-, 2-, 3-, and 5-year DFS rates for TNT were 86%, 78%, 67%, and 65%, respectively, while OS rates were 93%, 78%, 78.9%, and 74% [8]. The non-significant difference in terms of DFS, contrary to what was found for OS, can be attributed to the heterogeneity and methodological limitations of the included studies (non-randomized designs, variability in TNT sequencing and chemotherapy intensity, differences in follow-up), rather than a true biological dissociation between DFS and OS [8]. A more recent Australasian meta-analysis by Bedrikovetski et al. [15], analyzing 11 cohort studies and 8360 patients, found no significant differences among iTNT, cTNT, and standard CRT, although cTNT ranked highest for 5-year DFS and iTNT for 5-year OS. However, also in this metanalysis the heterogeneity and the methodological bias of the included studies represent a limitation [15].

Patient compliance with TNT is generally excellent, with CRT completion rates ranging from 81.9% to 100% [8]. When comparing TNT strategies, iTNT appears to be associated with higher rates of radiation-related gastrointestinal toxicity (OR: 5.12; CI: 1.82–17.6; *p* < 0.05) and postoperative diarrhea (OR: 2.71; CI: 1.37–5.67; *p* < 0.05)) and with lower rates of vomiting (OR: 0.240; CI: 0.0500–0.956; *p* < 0.05)) and lymphopenia (OR: 0.557; CI: 0.341–0.997; *p* < 0.05) [16].

Regarding surgical outcomes, TNT has demonstrated acceptable safety. In a meta-analysis by Lin. [17], no significant differences were found between TNT and standard CRT in 30-day postoperative mortality (0.50% vs. 0.68%), overall postoperative morbidity (35.2% vs. 32.9%), or major morbidity (14.5% vs. 13.9%) [17]. However, the TNT group showed a significantly higher risk of breached TME (7.2% vs. 4.8%), especially when treatment duration exceeded 17 weeks. Despite this, R0 resection rates (92.2% vs. 90.9%) and sphincter preservation rates (68.6% vs. 69.0%) were comparable between groups [17]. TNT was also not associated with a higher rate of disease progression (2.5% vs. 1.9%) [17]. Recently, an international multicenter study including 1585 consecutive patients from the RAPIDO, PRODIGE-23, and OPRA trials reported a TNT compliance rate of 97.9%, a treatment modification rate of 31.9%, a discontinuation rate of 13.5%, a 3-year DFS of 68%, and a 5-year OS of 79% [18].

The most consistent advantage of TNT is the significantly higher pCR rate. In the meta-analysis by Petrelli et al. [8], TNT increased the odds of pCR by 39%. In the PRODIGE-23 trial, pCR occurred in 27.8% of TNT patients compared with 12.1% after standard CRT [13]. Similarly, the RAPIDO and STELLAR trials reported significantly higher pCR rates after cTNT compared with standard CRT (28.4% and 21.8% vs. 14.3% and 12.2%, respectively) [11,19]. A network meta-analysis by Donnelly et al. [20] confirmed TNT as superior to standard CRT in achieving pCR, with cTNT producing the highest pCR rates [20]. The recent meta-analysis by Cheong et al. [21], including 33 studies (14 RCTs), also confirmed higher pCR rates with TNT; however, no significant differences were observed for cCR, OS, DFS, or local control [21].

## 3. Results of Organ-Sparing Approaches After TNT

The improvements in cCR and pCR achieved with TNT, together with the favorable oncologic outcomes associated with pCR, have led to an increased adoption of non-operative management (NOM) protocols (Table 1).

In the multicenter randomized RAPIDO trial, 912 patients with LARC were randomized (1:1) to receive either a TNT regimen with short-course RT (462 patients) or standard CRT (450 patients). In the TNT arm, 120 patients (28%) achieved a pCR—significantly higher than the 14% observed after standard CRT. Although NOM was not part of the study design, 25 patients (3%) were ultimately managed with a watch-and-wait (W&W) approach (14 in the TNT group and 11 in the CRT group). Among the TNT patients undergoing W&W, two (14%) developed distant metastases (vs. one, 9%, in the control group), and one (7%) developed local regrowth (vs. one, 9%) [11].

Similarly, in the PRODIGE 23 trial, NOM was considered a protocol deviation. Nonetheless, 29 patients with a cCR or near-cCR after neoadjuvant therapy proceeded to NOM (14 W&W and 15 LE), although no oncologic follow-up data were reported for this subgroup [13].

In the STELLAR trial, TNT with short-course RT and consolidation CT was compared with standard CRT. The combined rate of pCR and sustained cCR in the TNT group was 21.8%, significantly higher than the 12.3% observed with CRT. A total of 33 of 298 patients (11%) in the TNT group and 13 of 293 (4.4%) in the CRT group achieved a cCR; however, of the patients achieving cCR, 28/33 in the TNT group and 10/13 in the CRT group proceeded with NOM Local regrowth occurred in 2 of 28 (7.1%) TNT patients and 1 of 10 (10%) CRT patients. Long-term oncologic outcomes for these patients were not reported [19].

The first controlled trial specifically evaluating the safety of NOM after TNT was the OPRA trial. Patients with rectal cancer were randomized to iTNT (*n* = 146) or cTNT (*n* = 158) and, after restaging, were assigned to NOM (if cCR or near-cCR) or TME (if incomplete response) [22]. After restaging, the rate of cCR or near-cCR exceeded 70% in both groups (71.9% iTNT and 75.9% cTNT). However, the durability of response differed: in the cTNT group, 72.5% achieved a sustained cCR, while 27.5% developed local regrowth; in the iTNT group, these rates were 60% and 40%, respectively. Notably, 94% of regrowths occurred within two years, and all were managed with TME [22]. After a median follow-up of 5.1 years, the 5-year DFS rates were 71% (95% CI, 64 to 79) and 69% (95% CI, 62 to 77) for iTNT and cTNT, respectively (*p* = 0.068). TME-free survival was 39% (95% CI, 32 to 48) in the iTNT group and 54% (95% CI, 46 to 62) in the cTNT group (*p* = 0.012). Importantly, DFS was similar between NOM patients who experienced regrowth and subsequently underwent TME and those who underwent immediate TME after restaging [22]. Overall, the 3-year organ-preservation rate was 47%, significantly higher in patients with cCR than near-cCR (77% vs. 40%, *p* < 0.001) [23]. Furthermore, cCR status was strongly associated with DFS: 3-year DFS was 74% for the entire cohort but significantly higher in the cCR group (88% vs. 69% for near-cCR vs. 56% for incomplete responders, *p* < 0.001) [23].

Analogous efforts have been made to explore organ preservation in less advanced rectal tumors. The OPERA multicenter RCT investigated whether intensifying standard CRT could improve tumor response and enable W&W in patients with low–mid rectal cancer (cT2–3 a/b, <5 cm, < 50% circumference, cN0–1 with nodes < 0.8 cm, M0). Patients received CRT (45 Gy in 25 fractions with capecitabine 825 mg/m^2^) followed by either an external beam radiation therapy (EBRT) boost (9 Gy in 5 fractions; Arm A) or a contact X-ray brachytherapy (CXB) boost (90 Gy in 3 fractions; Arm B) [24]. The overall rate of cCR or near-cCR was significantly higher in Arm B (93%) than in Arm A (65%). Tumors < 3 cm responded particularly well, with cCR/near-cCR rates of 97% in Arm B versus 79% in Arm A (*p* = 0.04). The addition of CXB reduced local regrowth to 17% overall—much lower than the 39% observed in Arm A—and to only 3% in tumors < 3 cm. At 5-year follow-up, organ preservation was achieved in 79% of all patients and in 93% of those with tumors < 3 cm [24].

## 4. Criticism of the NOM Approach After TNT

The randomized OPRA trial introduced a new concept of NOM. In major NOM trials conducted prior to OPRA, the watch-and-wait strategy was adopted opportunistically when a cCR or near-cCR was detected after completion of CRT. The final decision to pursue NOM resulted from discussion with the patient regarding the feasibility and safety of avoiding surgery and undergoing intensive follow-up to allow for early detection of local regrowth or recurrence. In this context, the largest study evaluating the oncologic validity of NOM after cCR was the International Watch & Wait Database (IWWD), a multicenter retrospective study including 880 patients treated with NOM [25]. Among 667 (74.8%) patients with sustained cCR, the 5-year actuarial DFS and OS were 97.3% and 87.9%, respectively. Local regrowth occurred in 25.2% of cases and had a detrimental effect on oncologic outcomes: only 76% of regrowths were treated with completion TME, which achieved R0 resection in 88% of cases, while the 5-year DFS and OS (84.0% and 75.4%) were significantly worse compared with patients with sustained cCR [25].

Recently, the results from a retrospective multicenter study of 545 patients from the US Rectal Cancer Research Group were published. This study evaluated W&W outcomes in patients with cCR (initial or delayed) after TNT (72%) or standard CRT (28%). The overall oncologic outcomes were encouraging, with 3-year OS of 94.8% and 3-year DFS of 96.2%. However, local regrowth occurred in 84 (23.8%) patients, and 74 (88%) underwent salvage surgery (66 TME and 8 LE). Local regrowth was strongly associated with a higher incidence of distant metastases (14.2% vs. 3.5% in sustained cCR, *p* < 0.001) and negatively affected 3-year OS (83.6% vs. 97.7% in sustained cCR) [26].

Most of the available data derive from retrospective cohort studies, which are inherently prone to unmeasured confounding, selection bias, and heterogeneity in patient assessment and follow-up protocols. These limitations should be considered when interpreting the reported outcomes.

This approach entails several critical issues (Table 2). The first is the limited accuracy of restaging imaging, which prevents reliable confirmation of pCR. It is well known that cCR does not correspond to pCR in approximately 20% of cases [27]. Residual microscopic disease is the main cause of local regrowth, which occurs in approximately 20–25% of patients undergoing NOM [27]. Local regrowth requires salvage surgery—usually TME—with reduced likelihood of sphincter preservation [28]. Importantly, local regrowth has a negative impact on oncologic outcomes; in an ancillary IWWD analysis, it was identified as an independent predictor of worse distant metastasis-free survival [29].

In the OPRA trial, the watch-and-wait strategy was planned based on the high remission rates achieved with TNT, with the primary aim of organ preservation. Although high complete response rates partially justify this approach, significant concerns remain. The prognostic value of cCR has been well established. The British OnCoRe study [30], using propensity score matching, demonstrated that patients achieving cCR and undergoing NOM had significantly better 3-year OS compared with patients undergoing TME for residual disease (96% vs. 87%; *p* = 0.024). Similarly, in OPRA, the regrowth rate after cCR was 22%, significantly lower (*p*= 0.043) than after near-cCR (51%) or persistent tumor (63%). These differences corresponded to markedly different 3-year DFS rates of 88%, 69%, and 56%, respectively (*p* < 0.001) [23]. These findings highlight the risks of adopting NOM in near-cCR patients, who exhibit high rates of progression and DFS failure.

Local regrowth is the strongest predictor of distant metastasis. In a multicenter study of 793 NOM patients, distant metastases occurred in 24.1% of patients with local regrowth, compared with 5.8% of those with sustained cCR. This discrepancy may be attributed to intrinsically more aggressive tumor biology in regrowing lesions, as well as to a causal relationship in which residual treatment-resistant tumor cells serve as a source of dissemination [31].

This effect may be further amplified by the prolonged interval between initiation of TNT and surgery in patients with persistent, non-responding tumors (approximately 8 months in OPRA), potentially worsening the natural history of CRT-resistant disease [32,33,34,35]. Although local control after W&W is generally maintained if timely salvage TME is performed, the rate of definitive local failure after NOM is approximately 8%, as shown by meta-analysis—an important consideration when compared with outcomes after CRT followed by TME. Additionally, refusal of salvage TME further compromises outcomes and constitutes another limitation of the W&W strategy [43].

Another concern regarding TNT—and consequently NOM after TNT—is the prolonged interval between radiotherapy completion and surgery (typically 22–24 weeks), which is substantially longer than after standard CRT. In the RAPIDO trial, although TNT resulted in a significantly higher pCR rate compared with standard CRT (28% vs. 14%), the local failure rate was also higher (12% vs. 8%) [12]. These findings are probably related to the lower total biological radiation dose (short-course RT, 5 × 5 Gy) and due to repopulation of radioresistant and chemo resistant tumor clones, not fully eradicated by short course RT, during the extended interval between RT and response assessment [43].

This hypothesis is supported by an Italian retrospective study of 1064 patients comparing surgery performed within 8 weeks vs. after 8 weeks [44]. Longer intervals were associated with worse 10-year local failure (13.4% vs. 7.1%, *p* = 0.005) and worse 5-year DFS (59.6% vs. 72%, *p* < 0.001). These data suggest that TNT—by extending the interval between the start of therapy and surgery to increase cCR and near-cCR rates—may simultaneously increase the risk of distant metastases and local failure by leaving viable chemo resistant tumor cells in situ that would otherwise be removed sooner, as occurs with standard CRT [43]. Several trials enquiring the outcomes of NOM approach after TNT are ongoing. The mains are reported on Table 3.

A significant help to clinicians in rapidly identifying a local regrowth or local recurrence after NOM or organ-preserving approach could come from high-quality pelvic MRI. High-quality pelvic MRI, including diffusion-weighted sequences, represents an essential tool for early detection of residual or recurrent disease during surveillance following NOM and organ-preserving procedures TNT [45]. MRI-based follow-up allows for identification of subtle regrowth before clinical symptoms arise, improving the probability of effective salvage surgery and supporting the safety of organ-preservation strategies [45].

## 5. Role of Local Excision in cCR or Near-cCR Rectal Cancer Patients

Considering the limitations of NOM while preserving the organ-sparing philosophy, full-thickness LE of the rectal wall containing the scar or minimal residual tumor (cCR or near-cCR) has been introduced as the most effective diagnostic tool for confirming complete or major pathological tumor response (ypT stage) after CRT. If pCR or near-complete response (i.e., ypT0 TRG1 or ypT1 TRG2 with tumor-free margins) is confirmed, patients undergo close, intensive follow-up. Conversely, if histopathology does not demonstrate major or complete response and/or margins are involved, completion TME is recommended within one month of LE [36] (Table 2).

The GRECCAR 2 trial is the only randomized clinical trial comparing LE with radical TME in near-complete responders after standard CRT [37]. The study demonstrated the non-inferiority of LE compared with TME regarding oncologic outcomes, including 5-year LR (7% vs. 7%; *p* = 0.600), metastatic recurrence (18% vs. 19%; *p* = 0.730), DFS (70% vs. 72%; *p* = 0.680), OS (84% vs. 82%; *p* = 0.850), and cancer-specific mortality (7% vs. 10%; *p* = 0.530). Based on these results, the authors retrospectively analyzed 110 patients treated over 10 years. After 2022, completion TME for ypT2 tumors with favorable TRG2 was abandoned, resulting in a significant increase in 1-year organ preservation (from 63.3% to 91.8%; *p* < 0.01). After 3 years, DFS was comparable between groups (89.8% vs. 85.4%; *p* = 0.51), with one locoregional recurrence in each cohort [38].

The prospective multicenter Italian ReSARch trial evaluated the oncological feasibility of LE and W&W in 178 stage II–III rectal cancer patients treated with standard CRT achieving cCR or near-cCR. Among 107 cCR patients, 65 underwent W&W and 42 underwent LE. In the LE group, pCR was confirmed in 73.8%. Five patients showed high-risk features (ypT > 1, TRG > 2, or involved margins), and cTME was proposed in all but performed in four. LR occurred in 7.1%, all managed with salvage TME, and distant recurrence occurred in 4.8%. Rectal preservation was achieved in 80.9% (stoma-free rate 90.5%). In the W&W group, local regrowth occurred in 27.7%, treated with LE or cTME, with final rectal preservation of 81.5% and stoma-free survival of 87.7% [39].

Among 71 near-cCR patients, LE was performed in 70 and W&W in one. Fourteen patients required completion TME after LE, leading to an 80% rectum-preserving rate. LR and distant recurrence rates were 7.1% and 2.9%, respectively. Overall, combined W&W and LE achieved encouraging oncologic results (3-year OS: 97.6%; DFS: 90%; LR-free survival: 94.7%) and rectal preservation (3-year rectum-sparing rate: 80.6%; stoma-free survival: 95%) [39].

Organ-sparing LE also appears oncologically feasible in non-advanced low–mid rectal cancer. Management of early (T1–T2 N0 M0) rectal cancer remains controversial. Although TME ensures oncologic radicality, it carries significant risks of postoperative complications (e.g., anastomotic leak), long-term functional impairment (low anterior resection syndrome, urinary and sexual dysfunction), and stoma-related morbidity, raising concerns of overtreatment in many cases [46,47]. Conversely, transanal LE preserves the rectal reservoir and avoids such functional consequences but is generally considered oncologically safe only in selected low-risk T1 N0 tumors because of acceptable lymph node metastasis risks [48]. A recent meta-analysis of 3 RCTs and 27 observational studies (8570 patients) showed that radical surgery is associated with significantly lower positive margin and LR rates, although perioperative outcomes favor LE [40].

The introduction of neoadjuvant or adjuvant therapy may support LE as an alternative to TME in early high-risk cancers. In the first randomized trial on this topic, Lezoche et al. [49]. demonstrated non-inferior oncologic outcomes between neoadjuvant CRT followed by LE versus laparoscopic TME for early cT2 N0 “low-risk” cancers [49]. Similarly, a UK multicenter study of 60 cT1–2 N0 tumors treated with short-course RT followed by TEM reported major or pCR in two-thirds of patients and a 6.7% LR rate after 13 months, with all recurrences occurring in pT2 tumors [50]. In the CARTS study, LE after CRT achieved major or complete response (ypT0–1) in 30 of 47 patients, allowing for organ preservation, with only one LR after 17 months [51]. In a phase II trial (Kennecke et al. [52]), upfront chemotherapy allowed for organ preservation in 57% of patients, with promising locoregional relapse-free survival (98% at 1 year; 90% at 2 years) [52].

Unlike NOM, where decisions rely solely on clinical and radiologic findings, LE provides histological confirmation of ypT stage, allowing for identification of true pathological responders (Table 2). When LE confirms complete or major pathological response, rectal preservation is achieved with significantly better postoperative morbidity and functional outcomes than TME [7]. However, LE cannot assess nodal status with the same accuracy as TME. Nevertheless, nodal involvement is strongly correlated with ypT stage. The meta-analysis by Wee et al. [41], including 7568 TME-treated patients, reported a 4.6% nodal metastasis rate in ypT0 cases, consistent with other series [42]. This low risk supports the oncologic safety of organ preservation in ypT0 patients. In contrast, nodal involvement in ypT1 remains controversial, with some studies supporting safety [53,54] and others reporting >10% risk of nodal disease and higher recurrence rates [55].

By ensuring accurate assessment of residual tumor at the primary site, LE may reduce the risk of local failure and distant metastases linked to overlooked chemo-resistant residual disease in NOM.

Although LE provides histopathologic confirmation of ypT stage, several limitations must be acknowledged. Only one randomized controlled trial directly comparing LE with TME exists, but only in standard CRT setting; no randomized controlled trials about this comparative analysis exists in the setting of TNT. Full-thickness excision by LE cannot directly establish the pathological status of mesorectal nodes; however, it is indirectly possible to predict nodal status after CRT based on the yT stage. In a previous our series on 272 rectal cancer patients underwent to TME, the rate of positive nodes was 1.8% for pT0 and TRG1 cases and, at univariate analysis, clinical pre-treatment N stage (*p* < 0.05), pT stage (*p* < 0.001) and TRG (*p* < 0.001) were significantly correlated with the risk of lymph node metastasis [42]. This risk of positive nodes was reported also in the systematic review and meta-analysis of Wee et al. [41]. on 7568 patients treated with TME which reported a rate of nodal involvement of 4.6% in ypT0 patients [41]. A more criticism of LE is about the post-operative functional outcome. In our previous series, LE by TEM after CRT was significantly related to a higher incidence of LARS than not irradiated patients underwent to LE (41.2% vs. 10%, *p* = 0.012), even if the rate of major LARS in irradiated patients was 6.4% [56]. However, at 1 year from surgery, functional outcomes (evacuation and continence score) after LE and CRT seems to be significantly better than functional outcomes recorded after TME and CRT [7]. Another limitation of LE-based organ preservation is the potential need for completion TME (cTME) within 30 days when high-risk pathological features are present. cTME after LE in a previously irradiated pelvis is technically challenging and associated with increased APR rates, anastomotic leakage, morbidity, and inferior quality of life compared with primary TME [57,58]. These concerns have been partly mitigated by a recent Italian multicenter study—the largest series of cTME performed after LE and CRT—which evaluated 47 cases [59]. Abdomino-perineal resection (APR) rate (21.3%), minimally invasive surgery rate (59.6%), and R0 resection rate (95.7%) were acceptable. Postoperative morbidity was 34% (grade >2: 19.1%), with a 10.8% anastomotic leak rate. After median follow-up of 57 months, 89.7% underwent stoma reversal, resulting in a long-term stoma-free rate of 70.2%. Oncologic outcomes were favorable, with local and distant recurrence rates of 4.3% and 12.8%, respectively. Notably, residual tumor was found in 44.7% of patients—substantially higher than in GRECCAR 2 (10.7%) [51]—highlighting the necessity for cTME when adverse pathological features are present.

## 6. Conclusions

Although TNT has substantially increased pathological and clinical complete response rates, the evidence supporting the routine adoption of NOM remains limited, particularly in view of the high and clinically significant rate of local regrowth (>25%) and its negative prognostic implications. In this setting, LE could compensate for the mismatch between cCR and pCR by identifying and removing residual tumor foci that persist after TNT and could otherwise lead to local failure or distant metastasis both in the short and long term. Current prospective data seem to suggest that LE can offer an oncologically safer organ-preservation pathway than W&W in carefully selected cCR or near-cCR patients. However, the number of randomized trials specifically evaluating LE after TNT is still small, and additional high-quality prospective studies are needed before LE can be universally recommended as the preferred organ-preserving strategy in the TNT era.

Moreover, future research should aim to better define which patients are most likely to achieve sustained responses after NOM. A major unmet need is the identification of clinical, radiologic, pathological, and biological characteristics that distinguish long-term responders from patients at higher risk of local regrowth or distant recurrence. Actually, no clinically validated predictive model exists about these questions. Prospective studies integrating high-quality imaging, standardized response criteria, and translational biomarkers are therefore essential to develop robust predictive tools and improve patient selection for organ-preservation strategies.

## Figures and Tables

**Table 1:** Main trials on NOM after standard CRT and TNT for rectal cancer.

Trial	Study Design & Stage	TNT Regimen	Organ-Preservation Data	Main Findings Relevant to Organ Preservation
RAPIDO (2021) [11,12]	Phase III, LARC	SCRT + CapeOX/FOLFOX + TME	NOM allowed as deviation (*n* = 25)	TNT increases pCR (28% vs. 14% CRT). Limited NOM: regrowth 7%, metastasis 14%.
PRODIGE-23 (2021) [13]	Phase III, LARC	FOLFIRINOX + CRT + TME	29 NOM patients (14 W&W, 15 LE); no outcomes published	TNT increases pCR (27.8%). No structured NOM data.
STELLAR (2022) [19]	Phase III, LARC	SCRT + consolidation CT vs. CRT	28 NOM pts in TNT arm	Combined pCR + Sustained cCR 21.8% vs. 12.3%. Regrowth 7.1%.
OPRA (2024) [22,23]	Phase II RCT, LARC	iTNT vs. cTNT	Structured NOM strategy	cTNT yields higher organ preservation (54%). Regrowth: 27.5% (cTNT), 40% (iTNT).
OPERA (2023) [24]	Phase III, early T2–T3 low/mid	CRT + EBRT boost vs. CRT + CXB boost	Primary endpoint: organ preservation	CXB: higher cCR (93% vs. 65%), lower regrowth (17% vs. 39%), organ preservation 79–93% in tumors < 3 cm.

**Table 2:** Comparison between Watch-and-Wait and Local Excision after TNT.

Feature	Watch-and-Wait (W&W) [11,13,19,23,24,25,26,27,28,29,30,31,32,33,34,35]	Local Excision (LE) [36,37,38,39,40,41,42]
Primary goal	Avoid surgery (both TME than LE) in cCR	Confirm pCR/near-pCR while preserving rectum
Assessment of residual disease	Clinical + radiologic only	Histopathological confirmation
Removal of microscopic tumor	No	Yes
Local regrowth rate	20–30%	Risk of local recurrence of 5–10% in ypT0–1
Need for TME	High (70–80% of regrowth) risk of salvage TME	Only if adverse pathology is necessary a completion TME
Accuracy in predicting pCR	About 75%	100% for ypT stage
Impact on distant metastasis	Higher in regrowth	Lower in ypT0–1
Organ preservation rates	40–60%	70–90%
Functional outcomes	Excellent if sustained cCR	Excellent
Main limitations	High rate of regrowth and its related worse oncological outcomes. Misclassification	No information about ypN (only hypothetical risk related to ypT) Feasibility of cTME
Best candidates	Strict cCR	cCR or near-cCR with residual scar

**Table 3:** Main ongoing clinical trials assessing W&W after TNT or neoadjuvant therapy in rectal cancer.

Trial	Baseline Stage	TNT Regimen	W&W Eligibility	Primary Endpoint(s)	Status
Total neoadjuvant therapy followed by a watch-and-wait strategy for patients with rectal cancer (TOWARd) **RCTs031220288**	Early-stage cT2–3 N0 M0, tumour ≤ 5 cm	TNT; restaging q8 weeks post-TNT	cCR and W&W; near-cCR and W&W or LE; incomplete and TME	Phase II: cCR rate Phase III: 5-yr overall survival	Ongoing
Total Neoadjuvant Therapy and Organ Preservation Versus Surgery for Rectal Cancer **NCT06758830**	LARC	TNT	cCR after TNT	Organ-preservation rate; disease-control outcomes (per registry)	Ongoing
Total Neoadjuvant Therapy Followed by ‘Watch and Wait’ Approach or Organ Preservation for Low-risk Rectal Cancer (BJCC-R01) **NCT04405206**	Low-risk MRI-stratified rectal cancer	Intensity--modulated nCRT + capecitabine with consolidation CapeOX (TNT)	cCR/low-risk candidates for NOM	Efficacy of TNT and W&W/organ preservation	Active

## Abbreviations

The following abbreviations are used in this manuscript:

LARC	locally advanced rectal cancer
TME	total mesorectal excision
RT	radiotherapy
CRT	chemoradiation therapy
LR	local recurrence
pCR	pathological complete response
TNT	total neoadjuvant treatment
CT	chemotherapy
iTNT	induction TNT
cTNT	consolidation TNT
cCR	clinical complete response
NOM	non operative management
LE	local excision
DFS	disease-free survival
OS	overall survival
W&W	watch & wait
EBRT	external beam radiation therapy
APR	abdominoperineal resection
cTME	completion TME
